# Genetic susceptibility to methylmercury developmental neurotoxicity matters

**DOI:** 10.3389/fgene.2013.00278

**Published:** 2013-12-13

**Authors:** Jordi Julvez, Philippe Grandjean

**Affiliations:** ^1^Department of Environmental Health, Harvard School of Public HealthBoston, MA, USA; ^2^Centre for Research in Environmental EpidemiologyCatalonia, Spain; ^3^Department of Environmental Medicine, University of Southern DenmarkOdense, Denmark

**Keywords:** genetic variation, gene environment interactions, neurodevelopment, methylmercury, developmental toxicity, environmental epidemiology

Epidemiological studies have demonstrated the developmental neurotoxicity associated with prenatal methylmercury exposure (Grandjean and Landrigan, 2006); However, susceptibility to methylmercury toxicity may be increased by genetic factors. This observation raises the question of possible dependence of developmental neurotoxicity on genetic predisposition. A few years ago, a National Research Council (NRC) evaluation of the scientific background for risk assessment concluded that “attention should be directed to vulnerable individuals and subpopulations that may be particularly susceptible or more highly exposed” (National Research Council, 2009). The panel also noted that “variability in susceptibility and vulnerability has received less detailed evaluation in most EPA health effects assessments.” A previous NRC review estimated that, under certain circumstances, individual susceptibility could range up to 50,000-fold, and as much as 5% of the population could well be at least 25-fold more susceptible than the average (National Research Council, 2000).

In risk assessment, an uncertainty factor of 10 is commonly used to take into account intraspecies susceptibility. In regard to methylmercury, at the recommendation of the NRC (National Research Council, 2009), EPA used the default 10-fold intraspecies uncertainty factor. In contrast, the European Food Safety Authority recently argued that, for methylmercury, a partial uncertainty factor of 2 would be sufficient when a benchmark dose level (BMDL) had been obtained from a birth cohort that would represent the most vulnerable population (European Food Safety Authority, 2012). The magnitude of the intraspecies uncertainty factor therefore appears controversial, and better scientific documentation has been recommended (Dorne, 2010). Given that gene-environment interaction (GxE) may also play a role in regard to disease pathogenesis in a more general sense, as highlighted by a recent NIH workshop (Bookman et al., 2011), the variability in susceptibility to neurotoxicity between population groups appears to be an important research priority.

The recent findings on GxE for methylmercury neurotoxicity are supported by several previous studies. Thus, mutations in certain genes seem to convey a greater risk of elemental mercury-associated neurobehavioral deficits or symptoms in adults working in dental clinics (Echeverria et al., 2006, 2010; Heyer et al., 2008, 2009). This evidence was recently extended to children exposed to inorganic mercury from amalgam fillings (Woods et al., 2012, 2013). The reasons for such interactions are only partially understood, but some gene variants may predict a greater retention of mercury compounds in the body.

Thus, gene mutations seem to affect the retention of inorganic mercury and methylmercury in the body, e.g., genes that affect glutathione (GSH) and metallothionein metabolism (Gundacker et al., 2007; Schlawicke Engstrom et al., 2008; Wang et al., 2012). Other studies have also considered absorption-distribution-metabolism-elimination (ADME) genes that may be of importance. Thus, methylmercury is eliminated from the liver as GSH conjugates, and the rate-limiting enzyme for GSH synthesis is glutamyl-cysteine ligase (GCL), which is composed of a catalytic subunit (GCLC) and a modifier subunit (GCLM). Further, the glutathione-S-transferases (GST) catalyze the conjugation of GSH (Gundacker et al., 2007). A recent study in Sweden indicates that a *GCLC* polymorphism affects methylmercury retention, and that glutathione S-transferase pi 1 (*GSTP1*) may play a role in conjugating methylmercury with GSH (Schlawicke Engstrom et al., 2008). The GCLC SNP (Single Nucleotide Polymorphism) rs1555903 also showed a highly significant (*p* = 0.007) main effect on mercury retention in the umbilical cord in a UK birth cohort (Julvez et al., 2013). Mutations in glutathione S-transferase mu 1 (*GSTM1*) and *GCLM* also seem to affect the retention of methylmercury from fish and seafood (Barcelos et al., 2013).

Another possibility is that certain genotypes are less resistant to particular toxic effects. For example, in a study of dentists and dental assistants, deficits in neuropsychological performance occurred most frequently if the subject had a mutation in the coproporphyrinogen oxidase gene (*CPOX4*) (Echeverria et al., 2006); the same SNP also showed a significant main effect on general cognitive function in a UK birth cohort (Julvez et al., 2013). Recently, Woods et al. (Woods et al., 2012, 2013) published two reports about GxEs in regard to mercury exposure and child neurodevelopment. The findings showed that *CPOX4* rs1131857 multi-variant status modified the toxic effects of chronic mercury (Hg) exposure in urine samples of children and adolescents. Similarly, an increased frequency of symptoms occurred in those who had a mutation (rs4680) in a gene that codes for catechol O-methyltransferase (*COMT*) (Heyer et al., 2009). A different *COMT* mutation showed a significant main effect on methylmercury levels in the UK cohort (Julvez et al., 2013). Mutation of the gene responsible for formation of metal-binding metallothionein (*MT*) may also result in a predisposition to adverse effects from elemental mercury exposure in children (Woods et al., 2013). In the UK cohort, metallothionein 2A (*MT2A*) rs10636 showed a significant main effect on general cognitive functioning (Julvez et al., 2013).

A recent prospective study of 2-year old children suggested that apolipoprotein E (*APOE*) variants modified the adverse effects of cord blood mercury on neurodevelopment (Ng et al., 2013). The gene product, apolipoprotein E is a protein transporter expressed in the brain, and the Epsilon4 allele is associated with poor neural repair function and is a risk factor for Alzheimer disease, it has also been shown to affect the neurobehavioral toxicity of lead in adults (Stewart et al., 2002). In the UK birth cohort, the *APOE* rs405509 showed significant main effects on methylmercury levels (data not shown).

None of these studies focused on the potential environmental exposure of methylmercury during pregnancy and each study tested only a few SNPs, with the exception of our recent study (Julvez et al., 2013), which aimed to assess prenatal methylmercury (MeHg) exposure and genetic predisposition to cognitive deficit in children of the Avon Longitudinal Study of Parents And Children (Bristol, UK). A subsample of the cohort was selected, where mercury concentrations could be measured in freeze-dried umbilical cord tissue as a measure of methylmercury exposure. The potential modification effect of 66 genes was analyzed; these genes were related to four major biological pathways that are considered important to neurodevelopment or metal neurotoxicity: (a) brain development and neurotransmitter metabolism, (b) cholesterol metabolism, (c) iron regulation, (d) peroxidative defense and other miscellaneous pathways (Gundacker et al., 2010). The findings suggested that four SNPs (rs2049046, rs662, rs3811647, and rs1042838) functionally related to the Brain-Derived Neurotrophic Factor (*BDNF*), Paraoxonase 1 (*PON1*), Transferrin (*TF*) and Progesterone Receptor (*PGR*) genes appeared to modify the methylmercury-outcome associations with cognitive deficits in children with the minor alleles (mutations).

The gene-methylmercury interaction findings from these studies suggest several potential biological pathways may play a role in the toxicity action. *PON1* codes for an enzyme that inhibits oxidation of lipoproteins through hydrolysis of lipid peroxides. Such oxidative damage can be induced by methylmercury (Hernández et al., 2009; Ayotte et al., 2011). *BDNF*, *CPOX4*, and *PGR* are related to brain development and neurotransmitter metabolism and methylmercury could interact to their receptors and modify the beneficial and protective effects derived from those genes during neurodevelopment (Echeverria et al., 2006). Cord serum concentrations of the BDNF protein decrease at higher prenatal methylmercury exposures (Spulber et al., 2010). Finally another potential pathway suggested by *TF* gene is through iron uptake mechanism (Yokel, 2006). A neurotoxic effect could be due to an increased level of exposure passing the blood-brain barrier (Woods et al., 2013).

These interactions are of interest, as they suggest potential biological pathways that may have implications for our understanding of the mechanisms of action for developmental neurotoxicity due to methylmercury. However, there are also important implications in regard to risk assessment. In our study, any overall methylmercury toxicity was not detectable at the low average exposure levels (mean: 26 ng/g in umbilical cord, corresponding to about 0.5 μg/g hair), even when adjustments for beneficial dietary factors from maternal seafood intake and social class were included in the models. One might therefore assume that the developmental neurotoxicity was negligible. However, adverse associations among genetically susceptible groups were discovered in analyses that were stratified by the SNP allelic variants. While the wild type was associated with benefits from increased methylmercury exposure (as a marker of maternal seafood diet) one or two mutations in the genes led to lower IQs at age 8 years. Furthermore, the importance of such genetic predisposition is illustrated by the fact that 21 percent of the cohort subjects had at least four minor alleles in the four SNPs identified; this subgroup showed methylmercury-associated cognitive deficits with low *p*-values for interaction (*p*-values for interaction = 0.002 and 0.0001). Given that the apparent increase in IQ among those who had no more than one mutation in the four genes is likely due to the benefits from maternal seafood diets, then the much lower IQs in children with minor alleles are worrisome. In some analyses, the difference between the groups suggests that children with at least four mutations lose as much as 25 IQ point more than wild type children at a 10-fold increase in prenatal methylmercury exposure. This finding suggests that current exposure limits may be too lax for a sizable fraction of the general population. One caveat must be mentioned. As some of the candidate genes may show pronounced differences in allele frequencies between ethnic groups, adjustment for potential genetic confounding must be considered. Because of the genetic background of the English and immigration during recent decades, the ALSPAC cohort can be considered an admixed population. Heterogeneity of genetic background can potentially lead to spurious associations if ancestry is related to both a candidate gene and the disease outcome of interest, i.e., genetic confounding due to population stratification (Ziv and Burchard, 2003). In future studies, the risk of genetic confounding should be based on a set of ancestry informative markers to reveal latent population structures within the study. Estimated proportions can then be included in statistical models as covariates to adjust for potential genetic confounding. However, the poor correlation between mutations of the four genes identified in the ALSPAC study would speak against genetic confounding as an explanation. Thus, these findings should inspire an increased interest in GxE studies in regard to developmental neurotoxicity caused by mercury and, possibly, other environmental chemicals.

In regard to organochlorine compounds (OCs), Morales et al., observed that *GSTP1* Val105 polymorphism modified effects of prenatal p,p′-Dichlorodiphenyltrichloroethane (*p,p*′-DDT) exposure on cognitive functioning in preschoolers, thus suggesting oxidative stress as a potential neurotoxicity mechanism (Morales et al., 2008). The same *GSTP1* Val105 polymorphism conferred excess susceptibility to the cognitive effects of cumulative lead exposure in a Boston-based prospective cohort of men (Eum et al., 2013). The two reports suggest similar toxic pathways from lead and DDT exposures, but further research is required with a more complete set of candidate genes.

In conclusion, environmental neuroepidemiology studies need to include a new focus on genetically susceptible groups in order to assess a more realistic potential risk of neurotoxicant exposures at low levels. Meanwhile, the regulatory agencies and risk assessment professionals should consider a precautionary approach taking into account the likely existence of genetic predisposition to neurotoxicity.
